# Quality Preservation and Storage Stability of Mozzarella Cheese Using Active PLA/Tragacanth Gum Films Incorporated With *Zataria multiflora* Essential Oil

**DOI:** 10.1002/fsn3.72581

**Published:** 2026-11-11

**Authors:** Amirreza Hajjar Bargh, Negin Noori, Afshin Akhondzadeh Basti, Ali Khanjari, Hassan Gandomi, Ramin Khorrami, Asghar Azizian, Fereshteh Ghadami

**Affiliations:** ^1^ Department of Food Hygiene and Quality Control, Faculty of Veterinary Medicine University of Tehran Tehran Iran; ^2^ Department of Food Safety and Hygiene SR.C., Islamic Azad University Tehran Iran

**Keywords:** active packaging, aflatoxin inhibition, mozzarella cheese, shelf life, *Zataria multiflora* essential oil

## Abstract

This study evaluated the potential of active polylactic acid (PLA)/tragacanth gum films incorporating *Zataria multiflora* essential oil (ZEO) as a natural packaging approach to improve the quality and shelf life of mozzarella cheese during refrigerated storage. Mozzarella cheese samples were coated with PLA/tragacanth gum films containing different concentrations of ZEO (0.3%–0.9%), while untreated samples served as the control. The effects of these treatments on microbial quality, chemical stability, and sensory properties were evaluated during 9 days of refrigerated storage. In addition, an experimental challenge test was performed to assess the effect of the films on aflatoxin production by *Aspergillus parasiticus* at 25°C. Films containing ZEO significantly reduced microbial growth compared with the control. Total viable counts in samples treated with the 0.9% ZEO film were approximately 1.3 log CFU/g lower than the control and about 0.8 log CFU/g lower than the film without EO at the end of storage, and coliforms, presumptive *Staphylococcus aureus* , and molds were reduced by the active films (*p* < 0.05). In the challenge assay, aflatoxin B₁ production was markedly reduced (*p* < 0.05), decreasing from about 2060 μg/kg in the control to below 1000 μg/kg in samples packed with ZEO‐enriched films. Changes in pH followed a similar downward trend across all treatments, while the rise in peroxide value was significantly smaller in samples packaged with higher (0.6% and 0.9%) ZEO films (*p* < 0.05), remaining below 0.55 meq O_2_/kg cheese compared with 0.76 meq O_2_/kg cheese in the control. Sensory evaluation showed that ZEO incorporation significantly improved aroma, flavor, and overall acceptability (*p* < 0.05), while color and texture remained at acceptable levels without significant changes among treatments. Overall, PLA/tragacanth gum films containing ZEO represent an effective natural active packaging system for extending the shelf life and maintaining the quality of fresh mozzarella cheese.

## Graphical abstract

Active PLA/tragacanth films with *Zataria multiflora* essential oil delayed microbial spoilage and lipid oxidation, reduced aflatoxin production, and maintained sensory quality in mozzarella cheese.

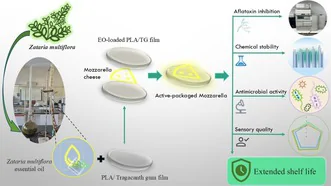

## 1 Introduction

The limited shelf life of fresh dairy products still represents a major challenge for the food industry. Products like mozzarella cheese need packaging strategies that can effectively delay spoilage while maintaining microbiological safety, physicochemical stability, and sensory quality. Because of its high moisture content and soft texture, mozzarella is especially vulnerable to quality deterioration during storage. Microbial growth, oxidation, and moisture transfer are the main factors driving its rapid spoilage, which can result in considerable economic losses (Huang et al. 2022). Consequently, developing effective packaging strategies capable of controlling microbial growth and oxidative deterioration has become an important priority for the dairy industry.

Although petroleum derived conventional plastics provide effective barrier properties, their environmental persistence and reliance on non renewable and environmentally harmful resources have raised major concerns regarding sustainability (Rajendran et al. 2025). So, recent research has shifted toward packaging solutions that balance effective product protection with environmental responsibility. This shift has led to important progress in developing sustainable, active, and intelligent packaging systems for better environmental outcomes (Abdullahi et al. 2025).

Biodegradable polymers have gained increasing attention as promising packaging materials. Among them, polylactic acid (PLA) has attracted considerable interest because it is bio‐based, compostable, and derived from renewable resources, while also offering good transparency and processability for food packaging applications (Mafe et al. 2025). However, pure PLA is brittle and exhibits limited barrier properties, which restrict its direct use in food packaging. To overcome these limitations, PLA is often blended with natural polymers (De Luca et al. 2023). Tragacanth gum (TG) serves as an anionic polysaccharide extracted from *Astragalus* species. It provides excellent film‐forming ability and rheological properties that enhance flexibility and moisture resistance in combination with PLA‐based films. Overall performance improves significantly through these enhancements (Bargh et al. 2025).

In addition to structural changes, packaging materials with active components improve food preservation. Active packaging embeds antimicrobial and antioxidant agents into the polymer matrix. These agents enable direct contact with food surfaces (Jasrotia et al. 2025). Essential oils (EOs) from plants serve as natural additives valued for bioactivity and consumer appeal. *Zataria multiflora* Boiss essential oil (commonly known as Shirazi thyme) possesses strong antimicrobial and antioxidant properties from phenolic compounds such as thymol and carvacrol. These compounds disrupt microbial cell membranes and inhibit oxidative reactions, making ZEO a promising natural preservative for extending the shelf life of high moisture foods such as fresh cheese. This essential oil, when integrated into biodegradable films, acts as a natural protective agent that reduces microbial growth and oxidative deterioration in packaged foods (Khajeh et al. 2024).

Despite previous research that characterized PLA/TG composite films containing *Zataria multiflora* essential oil (ZEO) in terms of their structural, physicomechanical, and antibacterial properties (Bargh et al. 2025), their practical performance in real food systems has not been sufficiently investigated. Therefore, this follow‐up study aimed to evaluate the application of PLA/TG active films incorporated with ZEO for preserving mozzarella cheese during refrigerated storage. In particular, the study examined their potential effects on microbial growth, aflatoxin production, lipid oxidation, and sensory attributes of the cheese, in order to assess their suitability as a natural active packaging system.

## 2 Material and Method

### 2.1 Material

All chemicals and solvents were of analytical grade. PLA was obtained from Greenjoy Biomaterial Co. Ltd., China. TG was supplied by Pakan bazr Company, Isfahan, Iran. ZEO was also procured from Pakan bazr Company. Mozzarella cheese was purchased from a commercial manufacturer. Culture media (including Plate Count Agar, Baird‐Parker agar, Violet Red Bile Agar, Brilliant Green Bile Broth, Yeast Extract Glucose Chloramphenicol agar, and Potato Dextrose Agar) were obtained from Merck, Germany, and Quelab, Canada. Tween 80 and analytical reagents for chemical analyses (including hydrochloric acid, ethanol, diethyl ether, sulfuric acid, sodium hydroxide, potassium iodide, sodium thiosulfate, and boric acid) were purchased from Merck, Germany, and Sigma‐Aldrich, USA. Analytical equipment included a digital pH meter (Ohaus ST3100, Switzerland), homogenizer (Heidolph Instruments, Germany), incubators, ovens, Mojonnier fat extraction flasks, hemocytometer, UV sterilization lamp, and RP‐HPLC system (Waters Alliance, USA). An aflatoxigenic wild isolate of *Aspergillus parasiticus* ATCC 56775 was sourced from the fungal collection of the Mycology Research Center at the University of Tehran (Tehran, Iran).

### 2.2 Experimental Design

The experiment followed a completely randomized design. Prior to the main analyses, the chemical composition of fresh mozzarella cheese, including moisture, fat, and protein contents, was determined. Films used for packaging and subsequent tests were produced with thicknesses ranging from 0.101 mm for the PLA/TG film to 0.107–0.116 mm for films containing 0.3%–0.9% ZEO, as previously measured (Bargh et al. 2025).

For aflatoxin determination, cheese slices were inoculated and packaged with sterile films using five treatments: control (without packaging), film without essential oil (EO), and films containing different concentrations of ZEO (0.3%, 0.6%, and 0.9%). The samples were incubated at 25°C for 10 days and analyzed on day 10.

For the shelf‐life study, cheese samples (approximately 30 g) were packaged using the same five treatments, ensuring complete surface coverage of the cheese with the films. All samples were stored under refrigerated conditions (4°C) for 9 days. Microbiological and chemical analyses were performed on days 0, 3, 5, 7, and 9. Sensory evaluation was conducted on day 9 using a 5‐point hedonic scale by 20 trained panelists to assess color, texture, taste, odor, and overall acceptability.

All analyses (except sensory evaluation) were performed in triplicate, and statistical analysis was conducted as described in the statistical analysis section.

### 2.3 Analysis of Key Components

#### 2.3.1 Determination of Fat Content

The total fat content of cheese samples was determined using a modified Mojonnier ether extraction method, as previously described for cheese analysis (Sabouri et al. 2024). Briefly, 5 g of finely grated cheese was placed into a Mojonnier fat extraction flask. Fifteen mL of hydrochloric acid was added in order to digest the protein matrix. Then, the mixture was vigorously shaken for 5 min. Afterwards, 15 mL of 95% ethanol was added to prevent protein precipitation. Fat extraction was then carried out using 45 mL of diethyl ether. The ether extract was collected in a flask (which was weighed before), and the solvent was evaporated at 100°C to a constant weight. After cooling in a desiccator, the flask was weighed, and the fat content was determined gravimetrically. The result was expressed as a percentage of the sample weight. All analyses were performed in triplicate.

#### 2.3.2 Determination of Protein Content

The protein content of the cheese samples was determined using the macro Kjeldahl method, which is a standard procedure for protein analysis in dairy products (Lynch and Barbano 1999). About 2 g of cheese samples were dried in an oven at 70°C for 5 h to remove moisture and then transferred to a Kjeldahl digestion flask for analysis. Concentrated sulfuric acid and a catalyst mixture of potassium sulfate, copper sulfate, and selenium oxide were added. The samples were digested until a clear solution was obtained.

After cooling, the digested samples were diluted with distilled water and made alkaline by adding 50% sodium hydroxide (NaOH). The released ammonia was distilled and collected in a boric acid solution containing a mixed indicator. The trapped ammonia was titrated with 0.1 N sulfuric acid to determine the total nitrogen content. Protein content was calculated by multiplying the nitrogen value by a conversion factor of 6.38, which is appropriate for cheese and milk products. All analyses were performed in triplicate, and the results were expressed as mean values.

#### 2.3.3 Determination of Moisture Content

The moisture content of cheese samples was determined using a gravimetric oven drying method, as described for cheese and cheese products (Bradley Jr and Vanderwarn 2001). Approximately 5 g of sample was accurately weighed into a glass dish (previously dried and weighed) and dried in a hot air oven at 103°C ± 2°C until constant weight was achieved. The dried samples were cooled in a desiccator and reweighed. Moisture content was calculated as the percentage of weight loss relative to the initial sample weight. All measurements were performed in triplicate.

### 2.4 Film Preparation

The PLA/TG composite films, with and without ZEO, were prepared identically to our previous method using the solution casting technique (Bargh et al. 2025). In brief, PLA and TG solutions were blended (1:1 ratio). For active films, ZEO (0.3, 0.6, or 0.9% v/v) was homogenized into the blend at 12,000 rpm for 2 min. All films were cast and dried at room temperature for 24 h. The prepared films were cut into uniform pieces and used to wrap mozzarella cheese samples, and this packaging procedure was applied consistently to all samples used for microbiological, chemical, and sensory analyses.

### 2.5 Preparation of Fungal Spore Suspension and Aflatoxin Assay

Mold cultures of *Aspergillus parasiticus* were grown on Potato Dextrose Agar (PDA) slants and incubated at 26°C ± 1°C for 7 days to promote sporulation. Spores were collected by adding 10 mL of sterile 0.05% (v/v) Tween 80 solution to each culture, gently scraping the surface, and filtering the suspension through sterile glass wool to remove mycelial debris. Spore concentration was determined using a hemocytometer and adjusted to 1 × 10^6^ spores/mL.

Mozzarella cheese (without preservatives) was aseptically obtained from a commercial manufacturer and surface‐sterilized using germicidal ultraviolet radiation. Cheese slices were placed in sterile Petri dishes and inoculated at the center with 10 μL of the spore suspension. The samples were then covered with sterile film and incubated at 25°C ± 1°C for 10 days.

After the incubation period, aflatoxins were extracted and determined according to Gandomi et al. (2009) with slight modifications (Gandomi et al. 2009). Briefly, each cheese sample was homogenized with 150 mL of methanol: water (80:20, v/v) and 2.5 g NaCl for 3 min, followed by defatting with *n*‐hexane. An aliquot of the extract was diluted with phosphate‐buffered saline (PBS), filtered through a glass‐fiber filter, and loaded onto an immunoaffinity column (Puri‐Fast AFLA IAC, Libios, France) at a flow rate of 3 mL/min. After washing the column with 15 mL of PBS and drying under a gentle stream of air, aflatoxins were eluted with methanol. The eluate was diluted with water, and 200 μL was injected into a reverse‐phase HPLC system (Waters 2695, USA) equipped with a Nova‐Pak C18 column and a fluorescence detector (Waters 474, USA). Post‐column derivatization was achieved using a Kobra Cell system (Libios, France) in a water‐acetonitrile‐methanol mobile phase containing HNO₃ and KBr at 1 mL/min (excitation: 365 nm, emission: 435 nm). Aflatoxin quantification was performed against certified analytical standards. The analytical method exhibited limits of detection of 0.4 μg/kg and limits of quantification (LOQ) of 0.9 μg/kg for all targeted aflatoxins. The method recovery rates were determined to be 82.9%, 85.8%, 86.7%, and 84.6% for AFB₁, AFB_2_, AFG₁, and AFG_2_, respectively.

### 2.6 Microbiological Analysis

Microbiological analyses were conducted to assess the antimicrobial effectiveness of the films during refrigerated storage at 4°C on days 0, 3, 5, 7, and 9. Total viable counts (TVC) were determined on Plate Count Agar (PCA) following incubation at 35°C for 48 h. Presumptive *Staphylococcus aureus* was enumerated on Baird‐Parker agar after incubation at 37°C for 48 h. Total coliforms were enumerated on Violet Red Bile Agar (VRBA) after incubation at 35°C for 24–48 h, with confirmation performed in Brilliant Green Bile Broth. Mold counts were determined on Yeast Extract Glucose Chloramphenicol (YGC) agar after incubation at 25°C for 5 days. Microbial populations were expressed as log CFU/g (Mileriene et al. 2021).

### 2.7 Chemical Analysis

#### 2.7.1 pH Measurement

The pH of the cheese samples was measured by homogenizing 10 g of sample with 90 mL of distilled water using a laboratory homogenizer. The pH of the homogenate was recorded at room temperature using a calibrated digital pH meter (Ohaus, model ST3100). Calibration was performed using standard buffer solutions at pH 4.0 and 7.0 prior to analysis. All measurements were conducted in duplicate, and results were reported as mean values (Sheerzad et al. 2024).

#### 2.7.2 Peroxide Value Determination

The peroxide value (PV) was determined using an iodometric titration method. Briefly, 3 g of the melted cheese sample (heated at 60°C for 3 min) was dissolved in 30 mL of an acetic acid‐chloroform mixture (3:2, v/v). The mixture was shaken for 3 min, followed by the addition of 0.5 mL of saturated potassium iodide (KI) solution. The liberated iodine was titrated with 0.01 N sodium thiosulfate (Na_2_S_2_O_3_) using starch as an indicator. PV was expressed as milliequivalents of active oxygen per kilogram of cheese (meq O_2_/kg cheese) (Sallam et al. 2004).

### 2.8 Sensory Evaluation

Sensory evaluation was conducted on day 9 of refrigerated storage by 20 trained panelists, including students and staff from the Veterinary Laboratory, University of Tehran. Cheese samples were cut into uniform portions (2 × 2 × 1 cm) and baked at 180°C for 10 min in an oven. The samples were then allowed to cool from the baking temperature to approximately 60°C (a suitable serving temperature to avoid consumer burns while preventing fat separation and preserving melt texture) prior to evaluation. All samples were coded with random numbers (3‐digit) and served in identical lidded containers to minimize bias. Panelists were blinded to the treatment identities and were not informed that the samples were from day 9 of storage. Panelists evaluated the samples for color, texture, taste, smell, and acceptability using a 5 point hedonic scale, where 1 represented extremely undesirable and 5 represented extremely desirable. Sensory responses were recorded using structured evaluation forms (Lawless and Heymann 2010). Participation of the panelists was voluntary, and informed consent was obtained from all participants prior to the sensory evaluation.

### 2.9 Statistical Analysis

Microbiological parameters (total viable counts, coliforms, *Staphylococcus aureus* , and molds) and chemical parameters (pH and peroxide value), evaluated during storage, were analyzed using two‐way analysis of variance (ANOVA) to assess the effects of packaging treatment (control, film without ZEO, and films containing 0.3%, 0.6%, and 0.9% ZEO), storage time, and their interaction. When significant differences were detected, mean comparisons were performed using Tukey's honestly significant difference (HSD) test. Aflatoxin concentrations, determined at a single storage time (day 10), were analyzed using one‐way ANOVA followed by Tukey's HSD test. Sensory evaluation data (assessed on day 9) were analyzed using the non‐parametric Friedman test to detect differences among treatments. For significant results (*p* < 0.05), post hoc pairwise comparisons were performed with Bonferroni correction. Statistical analyses were performed using SPSS software (version 22.0, USA) at a 95% confidence level, and results were expressed as mean ± standard deviation of independent replicates.

## 3 Results and Discussion

### 3.1 Chemical Composition of Mozzarella Cheese

The chemical composition of mozzarella cheese is presented in Table 1. The moisture content was 61.02% ± 0.74%, indicating that the product can be classified as a high‐moisture mozzarella cheese. The fat content was 19.10% ± 0.63%, while the protein content was 18.40% ± 0.81%, reflecting a nutritionally dense cheese matrix.

**TABLE 1:** Chemical composition of Mozzarella cheese on a wet‐weight basis (Mean ± SD, *n* = 3).

Chemical parameter	Content% (w/w)
Moisture	61.02 ± 0.74
Protein	18.40 ± 0.81
Fat	19.10 ± 0.63

The moisture and fat contents observed in the present study are consistent with reported compositional criteria for high moisture mozzarella cheese, in which high water retention and adequate fat‐in‐dry‐matter levels are essential for desirable functional and melting properties. According to the reference standards reported by Chen et al. (2009), mozzarella cheese with high moisture typically exhibits moisture contents above 55% and fat values within an acceptable range, in agreement with the results obtained in this study (Chen et al. 2009).

The protein content recorded in the present work is comparable to values reported by El Owni and Sana (2009) (El Owni and Sana 2009), who evaluated mozzarella cheese produced using different processing methods and reported similar protein levels. Minor variations in protein content among studies may be attributed to differences in milk composition, processing conditions, and curd handling practices (Chen et al. 2009). In addition, variations in analytical procedures and sample preparation may also contribute to slight variations in reported protein values (El Owni and Sana 2009).

### 3.2 Effect of Active Films on Aflatoxin Production by *Aspergillus parasiticus* on Mozzarella Cheese

The effect of different concentrations of *Zataria multiflora* essential oil (ZEO) incorporated into edible films on aflatoxin production by *Aspergillus parasiticus* in mozzarella cheese is presented in Table 2. The control samples, which were not treated with essential oil, exhibited the highest aflatoxin levels. In contrast, cheese samples coated with films containing ZEO showed a significant reduction in aflatoxin production, with the lowest concentrations observed in samples treated with 0.9% ZEO.

**TABLE 2:** Effect of essential oil incorporated into films on aflatoxin production (μg/kg) in Mozzarella cheese inoculated with *Aspergillus parasiticus* and incubated at 25°C for 10 days (*n* = 3).

Treatment	Aflatoxin B_1_ (μg/kg)	Aflatoxin B_2_ (μg/kg)	Aflatoxin G_1_ (μg/kg)	Aflatoxin G_2_ (μg/kg)
Control	2062.18 ± 17.77^a^	113.42 ± 3.77^a^	6949.50 ± 77.74^a^	284.93 ± 5.37^a^
Without EO	2053.68 ± 11.34^a^	112.31 ± 2.59^a^	6761.88 ± 68.25^b^	280.98 ± 4.58^a^
0.3% EO	1412.45 ± 12.17^b^	55.32 ± 3.64^b^	2802.22 ± 24.68^c^	76.59 ± 4.36^b^
0.6% EO	1120.75 ± 9.65^c^	30.16 ± 3.83^c^	1772.83 ± 19.83^d^	44.45 ± 2.53^c^
0.9% EO	924.74 ± 18.76^d^	22.49 ± 2.25^d^	1514.05 ± 13.46^e^	32.82 ± 2.16^d^

*Note:* Values are mean ± SD. Different superscript letters within each column indicate significant differences (*p* < 0.05).

As shown in Table 2, increasing the concentration of ZEO in the films resulted in a significant decrease (*p* < 0.05) in the production of all four major aflatoxins (AFB₁, AFB_2_, AFG₁, and AFG_2_). Aflatoxin B₁, the most toxic and carcinogenic aflatoxin, was markedly reduced from 2062.18 μg/kg in the control sample to 924.74 μg/kg in cheese samples treated with 0.9% ZEO. A similar inhibitory trend was observed for aflatoxins B_2_, G₁, and G_2_. Notably, aflatoxin G₁ levels were higher than B₁ across the treatments (approximately 3.4‐fold in the control). Such variation in the B₁‐to‐G₁ profile is not uncommon in *Aspergillus parasiticus*, as the differential synthesis of these mycotoxins can be modulated by growth conditions, substrate matrix, and the regulatory expression ratio of key biosynthetic genes (such as *aflS*/*aflR*) (Schmidt‐Heydt et al. 2010). Although active films markedly decreased aflatoxin levels compared to the control, residual AFB₁ concentrations remained above regulatory limits, indicating that the films mitigated toxin formation in this challenge model rather than rendering the inoculated cheese safe.

The reduction in aflatoxin production can be attributed to the strong antifungal and anti‐toxigenic properties of thyme EO. This plant EO is rich in phenolic compounds, particularly thymol and carvacrol, with carvacrol being the dominant component in the present study. These compounds have been widely described as potentially disrupting fungal cell membranes, interfering with key enzymatic activities, and downregulating secondary metabolite pathways, which may serve as a plausible explanation for the observed reduction in aflatoxin accumulation (Oliveira et al. 2020). Furthermore, earlier investigations on *Aspergillus parasiticus* treated with ZEO demonstrated that despite substantial initial mycotoxin levels (4181.87 and 8852.5 ppb for B_1_ and G_1_, respectively), the ZEO significantly suppresses fungal growth and downregulates the transcription of critical aflatoxin biosynthesis pathway genes (including *nor‐1*, *ver‐1*, *omt‐A*, and *aflR*) (Yahyaraeyat et al. 2013). The edible film matrix could provide a favorable vehicle to maintain active volatile compounds at the cheese interface, where sustained surface contact and possible progressive diffusion may support extended antifungal activity.

The findings of this study are consistent with previous reports. Lorán et al. (2022) demonstrated that various EOs significantly inhibited both mycelial growth and aflatoxin (B_1_, B_2_, G_1_, and G_2_) production by *A. parasiticus* (Lorán et al. 2022). Similarly, Burt (2004) reported that phenolic constituents of thyme EO effectively reduced mycotoxin synthesis by altering membrane permeability and mitochondrial function in fungal cells (Burt 2004). Rasooli et al. (2006) also showed that EOs rich in phenolic compounds significantly inhibited the growth of *Aspergillus* species (Rasooli et al. 2006).

Minor differences in the extent of aflatoxin reduction between the present study and previously published data may be attributed to variations in EO composition, fungal strain, food matrix, and application method. Factors such as EO chemotype, film formulation, cheese moisture content, and storage conditions are known to influence the antifungal and anti‐toxigenic effectiveness of plant EOs.

### 3.3 Microbiological Analysis

The evolution of microbial populations in mozzarella cheese samples during refrigerated storage is presented in Table 3. The Total Viable Count increased progressively in all samples throughout storage. The control sample showed the highest TVC, reaching 7.40 log CFU/g on day 9, indicating considerable microbial growth. In contrast, samples packed with ZEO incorporated films demonstrated an inhibitory effect. The strongest reduction was observed in the 0.9% ZEO treatment, which exhibited significantly lower TVC values at all storage times, reaching 6.08 log CFU/g on day 9. This reduction is conceivably associated with the presence of phenolic components, which are commonly hypothesized in literature to impair membrane integrity and cellular metabolism (Pajohi Alamoti et al. 2022; Ziaee et al. 2018). Coliforms were not detected (< 2 log CFU/g) in all samples on day 0. During storage, the control and packaged samples with pure films (without EO) showed a marked increase in coliform counts. In contrast, the incorporation of EO significantly suppressed coliform growth. Notably, samples treated with 0.6% and 0.9% EO maintained coliform counts significantly lower than other groups throughout storage (*p* < 0.05). The population of presumptive *Staphylococcus aureus* increased significantly in the control sample, reaching 5.05 log CFU/g at the end of storage. The packaging films containing ZEO effectively reduced the growth of these bacteria in a range from 0.82 to 1.59 log CFU/g compared to the control group. The 0.9% EO treatment showed the greatest inhibition, with significant differences with other groups especially after day 5 till the end of the experiment (*p* < 0.05). Mold growth followed a similar trend, with the control sample showing the highest counts during storage. Active films containing EO significantly inhibited mold development, especially at higher EO concentrations. On day 9, mold counts in samples treated with 0.9% EO were reduced by approximately 1.6 log CFU/g compared to the control group. The antifungal activity of *Z. multiflora* EO is consistent with previous findings and is mainly associated with its high content of oxygenated monoterpenes (Akhtari et al. 2022; Emami et al. 2024).

**TABLE 3:** Effect of PLA/tragacanth gum films containing different concentrations of Shirazi thyme essential oil (ZEO) on microbial counts (log CFU/g) of mozzarella cheese during refrigerated storage (9 days).

Microorganism	Treatment	Day 0	Day 3	Day 5	Day 7	Day 9
Total Viable Count	Control	2.08 ± 0.08^aE^	4.57 ± 0.20^aD^	5.67 ± 0.21^aC^	6.44 ± 0.16^aB^	7.40 ± 0.12^aA^
	Without EO	2.08 ± 0.08^aE^	4.42 ± 0.07^aD^	5.22 ± 0.11^aC^	6.28 ± 0.14^abB^	6.87 ± 0.17^aA^
	0.3% EO	2.08 ± 0.08^aD^	4.07 ± 0.10^bC^	4.65 ± 0.11^bB^	6.12 ± 0.05^bcA^	6.33 ± 0.25^bA^
	0.6% EO	2.08 ± 0.08^aE^	3.83 ± 0.04^cD^	4.31 ± 0.09^cC^	5.66 ± 0.05^cB^	6.21 ± 0.06^bA^
	0.9% EO	2.08 ± 0.08^aD^	3.68 ± 0.12^cC^	3.95 ± 0.14^dC^	5.14 ± 0.10^dB^	6.08 ± 0.27^bA^
Coliforms	Control	< 2	2.95 ± 0.06^aD^	3.55 ± 0.04^aC^	4.14 ± 0.06^aB^	4.86 ± 0.09^aA^
	Without EO	< 2	2.78 ± 0.05^bD^	3.25 ± 0.03^bC^	3.86 ± 0.04^bB^	4.07 ± 0.08^bA^
	0.3% EO	< 2	2.46 ± 0.11^cD^	3.01 ± 0.08^cC^	3.46 ± 0.07^cB^	3.95 ± 0.06^bA^
	0.6% EO	< 2	< 2	2.15 ± 0.04^dC^	3.25 ± 0.05^dB^	3.65 ± 0.07^cA^
	0.9% EO	< 2	< 2	2.12 ± 0.02^dC^	2.29 ± 0.06^eB^	2.79 ± 0.05^dA^
*Presumptive Staphylococcus aureus*	Control	< 2	2.65 ± 0.04^aD^	3.13 ± 0.07^aC^	4.15 ± 0.08^aB^	5.05 ± 0.04^aA^
	Without EO	< 2	2.60 ± 0.09^aD^	3.02 ± 0.06^aC^	3.68 ± 0.05^bB^	4.59 ± 0.05^bA^
	0.3% EO	< 2	2.32 ± 0.04^bD^	2.71 ± 0.04^bC^	3.38 ± 0.08^cB^	4.23 ± 0.06^cA^
	0.6% EO	< 2	2.25 ± 0.06^bD^	2.41 ± 0.05^cC^	3.04 ± 0.06^dB^	3.87 ± 0.06^dA^
	0.9% EO	< 2	2.12 ± 0.04^cD^	2.32 ± 0.05^cC^	2.72 ± 0.07^eB^	3.46 ± 0.11^eA^
Molds	Control	< 2	2.74 ± 0.05^aD^	3.22 ± 0.08^aC^	4.24 ± 0.09^aB^	5.14 ± 0.05^aA^
	Without EO	< 2	2.69 ± 0.10^aD^	3.11 ± 0.07^aC^	3.77 ± 0.06^bB^	4.68 ± 0.06^bA^
	0.3% EO	< 2	2.41 ± 0.05^bD^	2.80 ± 0.05^bC^	3.47 ± 0.09^cB^	4.32 ± 0.07^cA^
	0.6% EO	< 2	2.34 ± 0.07^bD^	2.50 ± 0.06^cC^	3.18 ± 0.07^dB^	3.96 ± 0.07^dA^
	0.9% EO	< 2	2.21 ± 0.05^cD^	2.41 ± 0.06^cC^	3.11 ± 0.08^dB^	3.55 ± 0.12^eA^

*Note:* Values are expressed as mean ± standard deviation (*n* = 3). Different superscript lowercase letters (a–e) within the same column indicate significant differences between treatments on the same storage day (*p* < 0.05), while different superscript uppercase letters (A–E) within the same row indicate significant differences among storage days within each treatment (*p* < 0.05). Values < 2 log CFU/g indicate microbial counts below the detection limit of the method. Day 0 values represent a common baseline shared across all treatments prior to film application.

The antimicrobial performance of the active PLA/Tragacanth gum films observed in this study is in agreement with previous reports on the application of EO based edible films for cheese preservation. Mahcene et al. (2021) reported that sodium alginate films incorporated with various EOs significantly reduced TVC, fecal coliforms, and yeasts in homemade cheese. They also reported completely inhibiting *Staphylococcus* spp. and molds during storage by utilizing these packages (Mahcene et al. 2021). Similarly, Hosseini et al. (2024) demonstrated that chitosan nanogels co‐encapsulating ZEO and nisin effectively suppressed coliforms and other spoilage microorganisms in Iranian white cheese, achieving microbial stability comparable to conventional preservatives (Hosseini et al. 2024). Although migration kinetics and residual EO levels within the cheese were not directly quantified in this study, sustained interfacial contact and potential gradual diffusion from the biopolymer matrix represent a plausible explanation for the prolonged microbial inhibition observed during storage.

### 3.4 Chemical Analysis

The pH values of mozzarella cheese samples exhibited a gradual decline over the 9‐day storage period across all treatments (Figure 1). Initial pH values on day 0 ranged from 5.54 to 5.64, with the control group showing a slightly higher baseline (5.64) compared to the film‐packaged samples (5.54–5.58), which reflects typical values for fresh mozzarella cheese. Over the storage period, the magnitude of pH decline was relatively similar across all groups. This decrease ranged from approximately 0.13 units in the control (from 5.64 to 5.51) to about 0.15 units in the 0.9% ZEO treatment (from 5.54 to 5.39). While statistically lower pH values were recorded in the ZEO‐treated samples from day 5 onward (*p* < 0.05), this difference largely reflects the initial baseline offset combined with the slight progressive acidification across treatments, rather than an active film‐induced acidification. In cheese, pH changes can also be influenced by factors such as microbial metabolism, proteolysis, and the buffering capacity of proteins during storage (Bansal and Veena 2024). Similar minor downward pH trends during storage have also been observed in cheeses packaged with active films containing plant essential oils (Kavas et al. 2015).

**FIGURE 1:**
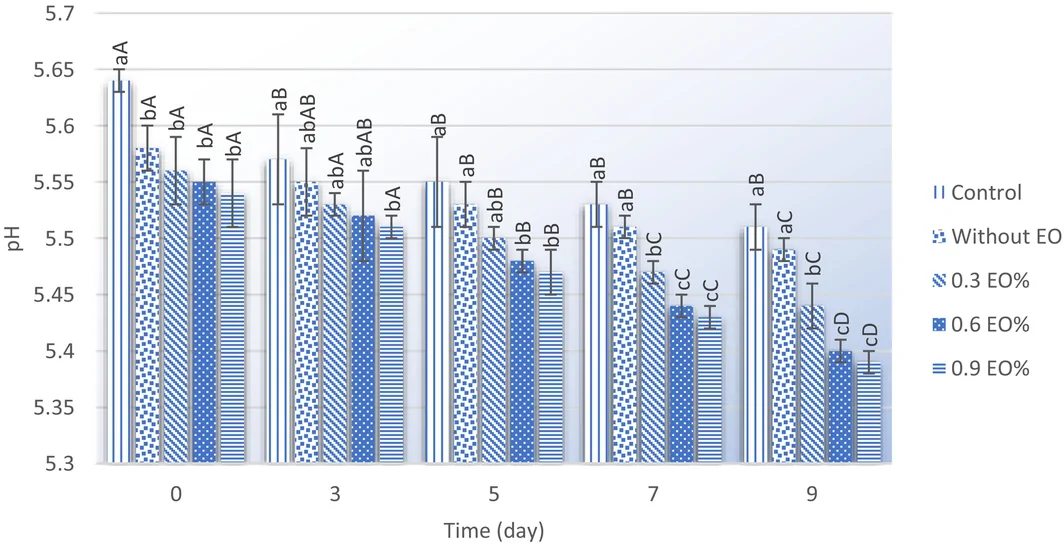
pH values of mozzarella cheese coated with PLA/tragacanth gum films containing different concentrations of Shirazi thyme essential oil (ZEO) during refrigerated storage (9 days). Different letters indicate significant differences (*p* < 0.05). Lowercase letters (a–c) represent differences among treatments at the same storage day, while uppercase letters (A–D) indicate significant differences during storage time within the same treatment.

Peroxide value (PV), as an indicator of primary lipid oxidation, remained low across all treatments throughout the storage time, reflecting the relatively short storage period and refrigerated conditions (Figure 2). Initial PV on day 0 was approximately 0.6 meq O_2_/kg cheese in the control, film without EO, and 0.3% ZEO groups, and about 0.5 meq O_2_/kg cheese in the 0.6% and 0.9% ZEO groups. By day 9, PV increased modestly to 0.76 meq O_2_/kg cheese in the control and film without EO, and to 0.65 meq O_2_/kg cheese in the 0.3% ZEO group. In contrast, higher ZEO treatments (0.6% and 0.9%) exhibited a noticeably smaller rise, stabilizing at approximately 0.52–0.53 meq O_2_/kg cheese. Statistical analysis revealed that from day 3 until the end of the experiment, PVs for samples packaged with films containing ZEO were significantly lower (*p* < 0.05) than for treatments without EO. Although absolute PV levels remained well within acceptable freshness limits across all groups, the more limited rise in hydroperoxides observed with 0.6% and 0.9% ZEO can be attributed to the phenolic compounds in ZEO, which scavenge free radicals and mitigate hydroperoxide accumulation (Ahmadi‐Dastgerdi et al. 2022). In another study on Gouda cheese using *Bunium persicum* EO, a similar suppression of peroxide formation during storage was noted (Saravani et al. 2019). These findings are in good accordance with our results.

**FIGURE 2:**
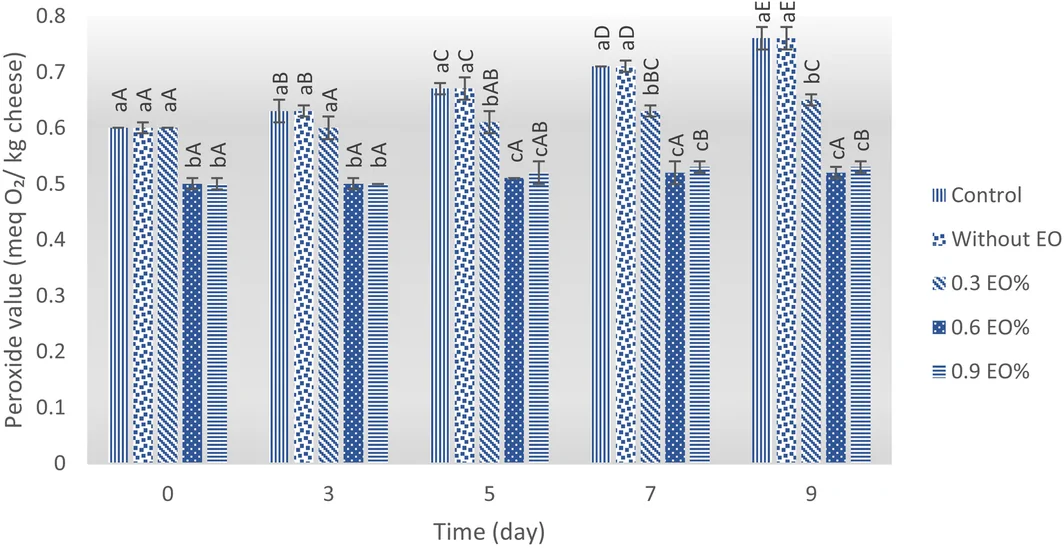
Peroxide value (PV, meq O_2_/kg cheese) of mozzarella cheese coated with PLA/tragacanth gum films containing different concentrations of Shirazi thyme essential oil (ZEO) during refrigerated storage (9 days). Different letters indicate significant differences (*p* < 0.05). Lowercase letters (a–c) represent differences among treatments at the same storage day, while uppercase letters (A–E) indicate significant differences during storage time within the same treatment.

### 3.5 Sensory Evaluation

The mean sensory scores for mozzarella cheese samples treated with different films after refrigerated storage are presented in Figure 3a–e. The incorporation of ZEO into PLA/Tragacanth gum films influenced several sensory attributes of the cheese samples. Statistical analysis revealed that no significant differences were observed among treatments for color and texture (*p* > 0.05). In contrast, significant differences were detected for taste, smell, and overall acceptability (*p* < 0.05). In general, samples packaged with ZEO‐containing films showed higher scores for these attributes compared with the control and the film without EO.

**FIGURE 3:**
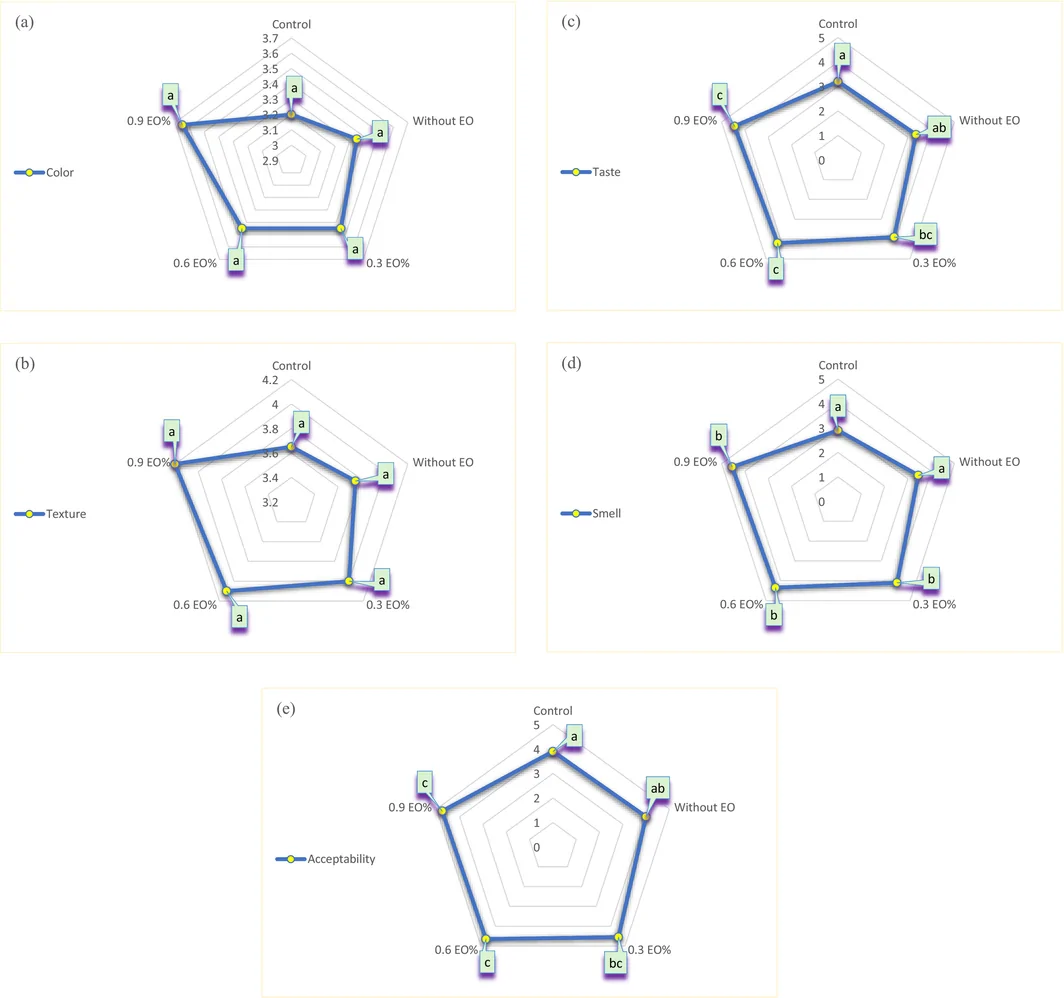
Sensory evaluation scores of mozzarella cheese coated with PLA/tragacanth gum films containing different concentrations of Shirazi thyme essential oil (ZEO) after 9 days of refrigerated storage: (a) color, (b) texture, (c) taste, (d) smell, and (e) overall acceptability. Different lowercase letters (a–c) shown on the charts indicate statistically significant differences among treatments (*p* < 0.05).

The improvement observed in taste and smell may be related to the antioxidant and antimicrobial activity of ZEO, which helped preserve the desirable sensory characteristics of mozzarella cheese during storage. As indicated by the microbial and chemical results, the presence of ZEO likely delayed microbial spoilage and lipid oxidation, processes that commonly lead to the development of off‐flavors and undesirable odors. The higher odor scores recorded for ZEO‐containing treatments support the ability of the essential oil to suppress or mask spoilage‐related volatile compounds. Similar positive sensory effects have previously been reported for active films incorporating plant essential oils and extracts (Cano Embuena et al. 2017). However, as sensory testing was conducted solely on day 9 without a baseline day‐0 evaluation, these ratings primarily indicate consumer preference rather than definitive preservation of initial sensory attributes.

Regarding texture, although slightly higher scores were observed in samples coated with active films, the differences among treatments were not statistically significant (*p* > 0.05). This observation may still be associated with the moisture‐retention capacity and barrier properties of PLA and tragacanth gum films, which can help maintain the characteristic soft and elastic structure of fresh mozzarella cheese (Bargh et al. 2025). Although samples containing higher concentrations of ZEO tended to receive slightly higher sensory scores, pairwise comparisons indicated no significant differences among the different ZEO concentrations (0.3%, 0.6%, and 0.9%).

Overall, the results suggest that the incorporation of ZEO into PLA/tragacanth gum films can impart favorable attributes such as taste, smell, and overall acceptability without negatively affecting color or texture, thereby enhancing the acceptability of fresh mozzarella cheese after refrigerated storage.

## 4 Conclusion

The findings of this study highlight the potential of PLA/tragacanth gum films enriched with *Zataria multiflora* essential oil (ZEO) as an effective natural preservation strategy for fresh mozzarella cheese. The incorporation of ZEO enhanced the antimicrobial and lipid‐stabilizing functionality of the packaging system. For total viable counts, reductions compared to the control and the film without EO ranged from 0.54 to 1.07 log CFU/g for the 0.3% ZEO film and from 0.79 to 1.32 log CFU/g for the 0.9% ZEO film. For mold populations, reductions ranged from 0.36 to 0.82 log CFU/g for the 0.3% film and from 1.13 to 1.59 log CFU/g for the 0.9% film compared to the control and film without EO. The ZEO‐incorporated films significantly outperformed both the control and the film without EO, noticeably moderating primary lipid oxidation with a smaller rise in peroxide values at higher essential oil concentrations (0.6% and 0.9% ZEO, maintaining levels below 0.55 meq O_2_/kg cheese), thereby contributing to the chemical stability and quality retention of the cheese during refrigerated storage. Furthermore, in a separate aflatoxin challenge assay (*Aspergillus parasiticus* inoculation at 25°C), the active films significantly suppressed aflatoxin synthesis, reducing AFB_1_ levels by up to ~55%, although residual toxin concentrations under this contamination model still exceeded regulatory limits.

In addition, sensory evaluation (on day 9) indicated that the active films, particularly those containing ZEO, were well accepted and contributed positively to aroma, flavor, and overall quality.

These results suggest that PLA/tragacanth gum films containing ZEO can serve as a promising natural active packaging approach for improving the storage stability and preserving the quality of high moisture cheeses such as mozzarella. However, the present study was conducted under controlled laboratory conditions and over a relatively short storage period. Therefore, further studies are required to evaluate the performance of these films during longer storage times and under industrial processing and distribution conditions. Future research should also focus on optimizing film formulation, investigating the release behavior of ZEO from the polymer matrix, and assessing the scalability and practical applicability of this packaging system in commercial dairy products.

## Author Contributions

**Amirreza Hajjar Bargh:** writing – original draft, investigation, data curation. **Negin Noori:** supervision, writing – review and editing. **Afshin Akhondzadeh Basti:** visualization, writing – review and editing. **Ali Khanjari:** writing – review and editing, conceptualization. **Hassan Gandomi:** methodology, data curation. **Ramin Khorrami:** validation, visualization. **Asghar Azizian:** investigation, writing – review and editing. **Fereshteh Ghadami:** investigation, writing – review and editing.

## Funding

The authors have nothing to report.

## Ethics Statement

This study was reviewed and approved by the Ethics Committee of the Faculty of Veterinary Medicine, University of Tehran, under the ethical approval code IR.UT.VETMED.REC.1405.008. The approval record is publicly available at: https://ethics.research.ac.ir/IR.UT.VETMED.REC.1405.008.

## Conflicts of Interest

The authors declare no conflicts of interest.

## Data Availability

Data sharing not applicable to this article as no datasets were generated or analysed during the current study.

## References

[fsn372581-bib-0001] Abdullahi, A. , T. Korumilli , and K. J. Rao . 2025. “Revolutionizing Food Packaging With Biobased Polymers, Active and Intelligent Materials for Enhanced Food Safety and Sustainability.” Food and Bioprocess Technology 18: 1–33.

[fsn372581-bib-0002] Ahmadi‐Dastgerdi, A. , N. Fallah , M. Zokaei , and M. Gholami‐Ahangaran . 2022. “The Role of Thyme (*Zataria multiflora* Boiss) Essential Oil as Natural Antioxidant on the Lipid Oxidation in Mayonnaise.” Journal of Food Quality 2022, no. 1: 1527289.

[fsn372581-bib-0003] Akhtari, A. , M. Davari , A. Habibi‐Yangjeh , A. Ebadollahi , and S. Feizpour . 2022. “Antifungal Activities of Pure and ZnO‐Encapsulated Essential Oil of *Zataria multiflora* on *Alternaria solani* as the Pathogenic Agent of Tomato Early Blight Disease.” Frontiers in Plant Science 13: 932475. 10.3389/fpls.2022.932475. PMC9294508 35865290

[fsn372581-bib-0004] Bansal, V. , and N. Veena . 2024. “Understanding the Role of pH in Cheese Manufacturing: General Aspects of Cheese Quality and Safety.” Journal of Food Science and Technology 61, no. 1: 16–26. 10.1007/s13197-022-05631-w. PMC10771476 38192705

[fsn372581-bib-0005] Bargh, A. H. , N. Noori , A. A. Basti , A. Khanjari , H. Gandomi , and R. Khorrami . 2025. “Multifunctional Polylactic Acid‐Tragacanth Gum Films Containing Shirazi Thyme Essential Oil (*Zataria multiflora* Boiss): A Structural and Functional Evaluation for Active Packaging.” Applied Food Research 5: 101454.

[fsn372581-bib-0006] Bradley, R. L., Jr. , and M. A. Vanderwarn . 2001. “Determination of Moisture in Cheese and Cheese Products.” Journal of AOAC International 84, no. 2: 570–592. 10.1093/jaoac/84.2.570. 11324627

[fsn372581-bib-0007] Burt, S. 2004. “Essential Oils: Their Antibacterial Properties and Potential Applications in Foods—A Review.” International Journal of Food Microbiology 94, no. 3: 223–253. 10.1016/j.ijfoodmicro.2004.03.022. 15246235

[fsn372581-bib-0008] Cano Embuena, A. I. , M. Cháfer Nácher , A. Chiralt Boix , et al. 2017. “Quality of Goat' s Milk Cheese as Affected by Coating With Edible Chitosan‐Essential Oil Films.” International Journal of Dairy Technology 70, no. 1: 68–76. 10.1111/1471-0307.12306.

[fsn372581-bib-0009] Chen, C. , D. Wolle , and D. Sommer . 2009. “Mozzarella.” In The Sensory Evaluation of Dairy Products, edited by S. Clark , M. Costello , M. Drake , and F. Bodyfelt , 459–487. Springer US.

[fsn372581-bib-0010] De Luca, S. , D. Milanese , D. Gallichi‐Nottiani , A. Cavazza , and C. Sciancalepore . 2023. “Poly (Lactic Acid) and Its Blends for Packaging Application: A Review.” Clean Technologies 5, no. 4: 1304–1343. 10.3390/cleantechnol5040066.

[fsn372581-bib-0011] El Owni, O. , and E. O. Sana . 2009. “Evaluation of Chemical Composition and Yield of Mozzarella Cheese Using Two Different Methods of Processing.” Pakistan Journal of Nutrition 8, no. 5: 684–687.

[fsn372581-bib-0012] Emami, M. , K. Zomorodian , S. Yazdanpanah , Y. Ghasemi , E. Mirzaei , and M. A. Derakhshan . 2024. “Multifunctional Bi‐Layer Collagen Nanofiber‐Collagen/PLLA/*Zataria multiflora* Essential Oil Nanofiber for Wound Healing: Antibacterial, Antifungal and Antioxidant Properties.” Journal of Drug Delivery Science and Technology 92: 105285. 10.1016/j.jddst.2023.105285.

[fsn372581-bib-0013] Gandomi, H. , A. Misaghi , A. A. Basti , et al. 2009. “Effect of *Zataria multiflora* Boiss. Essential Oil on Growth and Aflatoxin Formation by Aspergillus Flavus in Culture Media and Cheese.” Food and Chemical Toxicology 47, no. 10: 2397–2400. 10.1016/j.fct.2009.05.024. 19477213

[fsn372581-bib-0014] Hosseini, S. M. , H. Tavakolipour , M. Mokhtarian , and M. Armin . 2024. “Co‐Encapsulation of Shirazi Thyme (*Zataria multiflora*) Essential Oil and Nisin Using Caffeic Acid Grafted Chitosan Nanogel and the Effect of This Nanogel as a Bio‐Preservative in Iranian White Cheese.” Food Science & Nutrition 12, no. 6: 4385–4398. 10.1002/fsn3.4105. PMC11167143 38873443

[fsn372581-bib-0015] Huang, X. , F. K. Nzekoue , S. Renzi , et al. 2022. “Influence of Modified Governing Liquid on Shelf‐Life Parameters of High‐Moisture Mozzarella Cheese.” Food Research International 159: 111627. 10.1016/j.foodres.2022.111627 35940766

[fsn372581-bib-0016] Jasrotia, S. , S. Gupta , M. L. Kudipady , and Y. M. Puttaiahgowda . 2025. “Advancing Food Preservation With Quercetin‐Based Nanocomposites: Antimicrobial, Antioxidant, and Controlled‐Release Strategies‐A Review.” Current Research in Food Science 11: 101159. 10.1016/j.crfs.2025.101159. PMC12363476 40837384

[fsn372581-bib-0017] Kavas, G. , N. Kavas , and D. Saygili . 2015. “The Effects of Thyme and Clove Essential Oil Fortified Edible Films on the Physical, Chemical and Microbiological Characteristics of Kashar Cheese.” Journal of Food Quality 38, no. 6: 405–412. 10.1111/jfq.12157.

[fsn372581-bib-0018] Khajeh, N. , A. Mohammadi Nafchi , and L. Nouri . 2024. “Antioxidant and Antimicrobial Activities of Tarragon and *Zataria multiflora* Boiss Essential Oils and Their Applications as Active Agents to Improve the Shelf Life of Freshly Cut Potato Strips.” Food Science & Nutrition 12, no. 5: 3282–3294. 10.1002/fsn3.3995. PMC11077239 38726437

[fsn372581-bib-0019] Lawless, H. T. , and H. Heymann . 2010. Sensory Evaluation of Food: Principles and Practices. Springer Science & Business Media.

[fsn372581-bib-0020] Lorán, S. , J. J. Carramiñana , T. Juan , A. Ariño , and M. Herrera . 2022. “Inhibition of *Aspergillus parasiticus* Growth and Aflatoxins Production by Natural Essential Oils and Phenolic Acids.” Toxins 14, no. 6: 384. 10.3390/toxins14060384. PMC9227641 35737045

[fsn372581-bib-0021] Lynch, J. M. , and D. M. Barbano . 1999. “Kjeldahl Nitrogen Analysis as a Reference Method for Protein Determination in Dairy Products.” Journal of AOAC International 82, no. 6: 1389–1398. 10.1093/jaoac/82.6.1389. 10589493

[fsn372581-bib-0022] Mafe, A. N. , G. I. Edo , A. B. Ali , et al. 2025. “Next‐Generation Biopolymers for Sustainable Food Packaging: Innovations in Material Science, Circular Economy, and Smart Technologies.” Food and Bioprocess Technology 18, no. 11: 9052–9108.

[fsn372581-bib-0023] Mahcene, Z. , A. Khelil , S. Hasni , F. Bozkurt , M. B. Goudjil , and F. Tornuk . 2021. “Home‐Made Cheese Preservation Using Sodium Alginate Based on Edible Film Incorporating Essential Oils.” Journal of Food Science and Technology 58, no. 6: 2406–2419. 10.1007/s13197-020-04753-3. PMC8076434 33967337

[fsn372581-bib-0024] Mileriene, J. , L. Serniene , M. Henriques , et al. 2021. “Effect of Liquid Whey Protein Concentrate–Based Edible Coating Enriched With Cinnamon Carbon Dioxide Extract on the Quality and Shelf Life of Eastern European Curd Cheese.” Journal of Dairy Science 104, no. 2: 1504–1517. 10.3168/jds.2020-18732 33309377

[fsn372581-bib-0025] Oliveira, R. C. , M. Carvajal‐Moreno , B. Correa , and F. Rojo‐Callejas . 2020. “Cellular, Physiological and Molecular Approaches to Investigate the Antifungal and Anti‐Aflatoxigenic Effects of Thyme Essential Oil on *Aspergillus flavus* .” Food Chemistry 315: 126096. 10.1016/j.foodchem.2019.126096. 31991259

[fsn372581-bib-0026] Pajohi Alamoti, M. , B. Bazargani‐Gilani , R. Mahmoudi , et al. 2022. “Essential Oils From Indigenous Iranian Plants: A Natural Weapon vs. Multidrug‐Resistant *Escherichia coli* .” Microorganisms 10, no. 1: 109. 10.3390/microorganisms10010109 PMC8781614 35056560

[fsn372581-bib-0027] Rajendran, S. , A. Al‐Samydai , G. Palani , et al. 2025. “Replacement of Petroleum Based Products With Plant‐Based Materials, Green and Sustainable Energy—A Review.” Engineering Reports 7, no. 4: e70108.

[fsn372581-bib-0028] Rasooli, I. , M. B. Rezaei , and A. Allameh . 2006. “Growth Inhibition and Morphological Alterations of Aspergillus Niger by Essential Oils From Thymus Eriocalyx and Thymus x‐Porlock.” Food Control 17, no. 5: 359–364. 10.1016/j.foodcont.2004.12.002.

[fsn372581-bib-0029] Sabouri, N. , Y. Yamini , M. R. Ehsani , and H. Bakhoda . 2024. “Determination of Volatile Compounds in White Brine Cheese and Ultrafiltered Cheese During Ripening and Shelf‐Life Using Nano‐Adsorbent Fibers.” Journal of Food Science and Technology 61, no. 3: 573–584. 10.1007/s13197-023-05865-2. PMC10844181 38327853

[fsn372581-bib-0030] Sallam, K. I. , M. Ishioroshi , and K. Samejima . 2004. “Antioxidant and Antimicrobial Effects of Garlic in Chicken Sausage.” LWT‐Food Science and Technology 37, no. 8: 849–855. 10.1016/j.lwt.2004.04.001. PMC1805705 17330154

[fsn372581-bib-0031] Saravani, M. , A. Ehsani , J. Aliakbarlu , and Z. Ghasempour . 2019. “Gouda Cheese Spoilage Prevention: Biodegradable Coating Induced by Bunium Persicum Essential Oil and Lactoperoxidase System.” Food Science & Nutrition 7, no. 3: 959–968. 10.1002/fsn3.888. PMC6418427 30918638

[fsn372581-bib-0032] Schmidt‐Heydt, M. , C. E. Rüfer , A. Abdel‐Hadi , N. Magan , and R. Geisen . 2010. “The Production of Aflatoxin B1 or G1 by *Aspergillus parasiticus* at Various Combinations of Temperature and Water Activity Is Related to the Ratio of aflS to Afl R Expression.” Mycotoxin Research 26, no. 4: 241–246. 10.1007/s12550-010-0062-7. 23605486

[fsn372581-bib-0033] Sheerzad, S. , R. Khorrami , A. Khanjari , H. Gandomi , A. A. Basti , and F. Khansavar . 2024. “Improving Chicken Meat Shelf‐Life: Coating With Whey Protein Isolate, Nanochitosan, Bacterial Nanocellulose, and Cinnamon Essential Oil.” Lwt 197: 115912. 10.1016/j.lwt.2024.115912.

[fsn372581-bib-0034] Yahyaraeyat, R. , A. Khosravi , D. Shahbazzadeh , and V. Khalaj . 2013. “The Potential Effects of *Zataria multiflora* Boiss Essential Oil on Growth, Aflatoxin Production and Transcription of Aflatoxin Biosynthesis Pathway Genes of Toxigenic *Aspergillus parasiticus* .” Brazilian Journal of Microbiology 44, no. 2: 649–655. 10.1590/s1517-83822013000200045. PMC3833170 24294264

[fsn372581-bib-0035] Ziaee, E. , M. Razmjooei , E. Shad , and M. H. Eskandari . 2018. “Antibacterial Mechanisms of *Zataria multiflora* Boiss. Essential Oil Against *Lactobacillus curvatus* .” Lwt 87: 406–412. 10.1016/j.lwt.2017.08.089.

